# Bisphosphonate and statin: adverse effects of co-medication on wound healing in *in vitro* models of periodontal tissues

**DOI:** 10.2340/aos.v85.45585

**Published:** 2026-03-18

**Authors:** Agnes Småland-Reksten, Anne E. Agger, Aina M. Lian, Janne E. Reseland, Tormod B. Krüger

**Affiliations:** a Department of Oral Surgery and Oral Medicine, Faculty of Dentistry, University of Oslo, Oslo, Norway; b Department of Biomaterials, Faculty of Dentistry, University of Oslo, Oslo, Norway; c Oral Research Laboratory, Faculty of Dentistry, University of Oslo, Oslo, Norway

**Keywords:** Bisphosphonate, alendronate, statin, simvastatin, osteonecrosis

## Abstract

**Objective:**

Bisphosphonates and statins may influence wound healing and are frequently prescribed to the same patient group. Bisphosphonates may induce osteonecrosis of the jaw; however, little information is available on the cellular mechanisms and biological effects of co-medication in oral tissues. The aim was to assess the effects of alendronate and simvastatin, both alone and combined, on osteoblasts and gingival fibroblasts *in vitro*.

**Study design:**

Primary human gingival fibroblasts and primary human osteoblasts were incubated with alendronate (5 μM) and simvastatin (1, 5 or 10 μM), alone or combined, for up to 14 days. Cells were assessed for viability by measuring lactate dehydrogenase activity and caspase-3 concentration in the cell culture media. Migration and proliferation potential was assessed by scratch-wound assay. Secreted levels of cytokines/chemokines were measured using Luminex 200 multianalytic profiling.

**Results:**

High concentrations of simvastatin, both alone and combined with alendronate, affected the proliferation/migration potential and reduced scratch closure. The same exposure induced near abolishment of secreted levels of cytokines affecting angiogenesis, such as VEGF, IL-6, IL-8, and MCP-1, however little effect was found on cell viability.

**Conclusion:**

High concentrations of simvastatin, alone or combined with alendronate, may have a negative impact on angiogenetic markers and cell migration/proliferation, affecting wound healing and growth.

## Introduction

Bisphosphonates (BPs) are one of the most commonly used medications for treatment of osteoporosis, and in 2021 5.9% of the Norwegian population aged 60 years or more had at least one prescription of BPs, 99% of these being alendronate (ALN) [1]. Around 30% of the population aged ≥ 65 years in developing countries take five or more medications on a regular basis [2]. The patients treated with BPs for osteoporosis are generally a part of this age group and are thus likely to be treated for other diseases or conditions.

BPs main effect is inhibition of osteoclasts and thereby the reabsorption of bone. It has been shown that BPs also affect the growth of osteoblasts and have negative impact on vitality and migration of fibroblasts [3, 4].

Statin therapy, generally prescribed to reduce the risk of cardiovascular mortality and cardiovascular disease [5], has in some cases been reported to have a positive effect on wound healing. In a study where osteonecrosis of the jaw (ONJ) was present in rats, local injections of the statin Fluvastatin showed increased healing of the wound [6]. Simvastatin (SIM), one of the most commonly used statins in clinical practice and by 2.8% of the Norwegian population in 2021 [1], has been shown to increase bone formation [7–9], as well as having a healing effect on wound healing [10–12]. However, at higher concentrations it appears that it may have a negative impact on critical cell functions, which in turn may affect tissue repair [8, 13]. So far, the serum or tissue levels of SIM during treatment are unknown.

The current knowledge of the combined cellular effects of ALN and SIM is scarce, especially considering their widespread use, and correspondingly even less is found about their potential combined effect in oral tissues. The fact that BPs can have side effects, such as ONJ, where both bone and soft tissue are affected, underlines the importance of increased knowledge. There is no clear connection between SIM use and potential side effects such as ONJ, but in combination with other medications this cannot be ruled out. The aim of this study was therefore to assess the combined effect of ALN and SIM on gingival fibroblasts and primary osteoblasts in terms of viability, proliferation, secreted cytokines, and cell migration.

## Materials and methods

### Experimental set up

Primary gingival fibroblasts at passage 5 (HFIB-G; Provitro GmbH, Charitéplatz 1, 10117 Berlin, Germany) were seeded in culture plates at a density of 2.7 × 10^5^ (Wound scratch assay) and 3 × 10^5^ (LDH, Caspase-3 and Luminex). Growth medium (Dulbecco’s Modified Eagle’s Medium [DMEM] high glucose; Sigma-Aldrich, St. Louis, MO, USA) to which 1% GlutaMAX (35050; Gibco, Waltham, MA, USA), 10% fetal bovine serum (FBS) (F9665; Sigma-Aldrich) and 100 U/mL penicillin and 100 μg/mL streptomycin (15140-122; Gibco, Waltham, MA, USA) was added, and incubated in a humidified atmosphere at 37°C with 5% CO2.

Primary human osteoblasts from tibia of a 1-day old female donor at passage 4 (Cambrex BioScience, Walkersville, MD, USA) were grown in Lonza Osteoblast Growth Media (OGM) (Cambrex BioScience), containing ascorbic acid, fetal calf serum and gentamicin.

Both cell types were cultured to confluence and exposed to concentrations of alendronate (ALN 5 μM; Sigma-Aldrich Biotechnology, Saint Louis, MO, USA) and simvastatin (SIM 1, 5 or 10 μM; Sigma-Aldrich Biotechnology), alone or in combination. Medium was harvested and cell morphology and cell growth potential were evaluated for up to 14 days.

The chosen ALN concentration was based on research on therapeutically relevant concentrations [4]. For SIM, 1–5 μM seem to be the concentrations most referred to in previous articles [7, 13].

### Proteins secreted to cell culture medium

Cells were exposed to alendronate (5 μM) and simvastatin (1, 5 or 10 μM) and cell culture media was harvested after 1, 3, 7 and 14 days of incubation, with the latest medium change 24 h prior to harvest.

The cell culture media was evaluated for lactate dehydrogenase (LDH) using a cytotoxicity detection kit (11644793001; Roche, Basel, Switzerland). Aliquots of 50 μl media were mixed with 50 μl of the reactant mixture and incubated at room temperature for 30 min. BioTek ELx800 Absorbance Microplate Reader (BioTek instruments, Inc., Vermont, USA) was used to measure the absorbance at 490 and 690 nm.

Caspase-3 concentrations in the cell culture media were determined by the human Caspase-3 ELISA Kit (ab285337; Abcam Limited, Cambridge, UK) using a BioTek ELx800 Absorbance Microplate Reader (BioTek).

Multianalytic profiling of concentration of selected cytokines/chemokine secreted to the cell culture media was done using the Luminex 200TM system (Luminex Corporation, Austin, TX, USA) and the XY-platform. The fluorescence data were analyzed using the 3.1 xPONENT software (Luminex). The concentrations of cytokines and chemokines in the supernatants were determined using the 8-Milliplex Human Cytokine Magnetic Bead Panel (Millipore, Billerica, MA, USA). Included among the tested cytokines and chemokines were granulocyte colony-stimulating factor (G-CSF), granulocyte-macrophage colony-stimulating factor (GM-CSF), interleukin-1a (IL-1a), IL-6, IL-8, monocyte chemoattractant protein-1 (MCP-1), tumor necrosis factor-α (TNF-α) and vascular endothelial growth factor (VEGF). All analyses were performed according to manufacturers’ protocol.

### Wound scratch assay

Proliferation and migration were measured in confluent cell cultures scratched perpendicularly with a sterile 200 μL pipette tip. Cells were washed twice with PBS before adding SIM and/or ALN (set as timepoint 0 h). Various concentrations of SIM (1, 5 or 10 μM), alone or in combination with ALN (5 μM), were added to separate wells in triplicates. Cells incubated with cell culture media were used as control at each time point. Images of the cells were taken at 0, 4, 8, 12, 24 and 48 h using an Olympus IX70 light microscope (Olympus Corporation, Tokyo, Japan) with 1.25× magnification and the cell-free monolayer gap areas in the scratch were estimated using Fiji software (NIH, Bethesda, MD, USA). The area of the scratched opening in the confluent cell culture was calculated relative to the area at time point 0 h at different timepoints.

### Statistical analysis

Statistical analysis was performed using SigmaPlot version 15.0 (Systat Software, San Jose, CA, USA) and all results, except for the wound scratch assay, were calculated compared to unexposed controls at each time point. Student’s t-test, Mann–Whitney Rank Sum Test and P-value set to 0.05 was used to assess statistical significance. Not all data passed for normality and equality but were still included in the results.

## Results

### Viability, proliferation, and migration in fibroblasts

**Figure 1:**
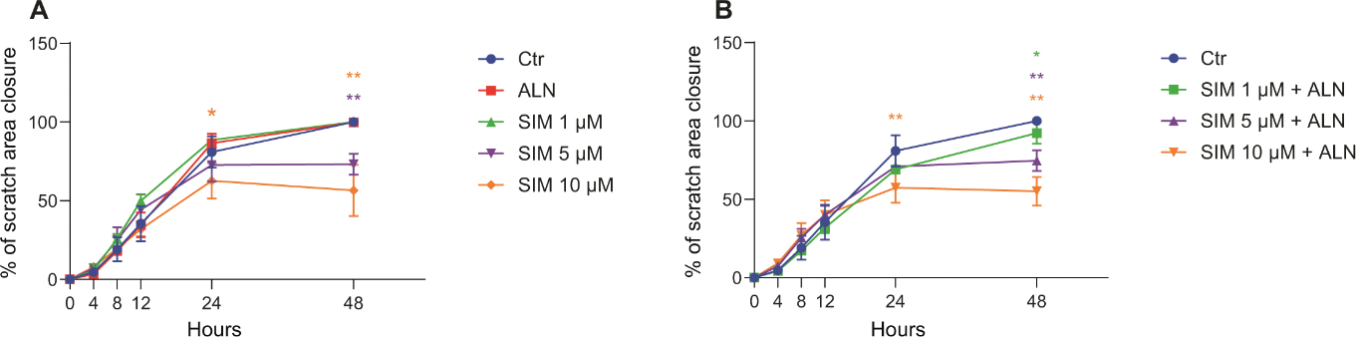
Scratched area closure of cells exposed to SIM (1, 5 or 10 μM), both alone (A), and in combination with ALN (5 μM) (B), presented in percentage of closure of the area created at timepoint 0 h. Significance compared to control is indicated with ***p < 0.001, **p < 0.01 and *p < 0.05, analyzed by t-test or Mann–Whitney Rank Sum Test when normality and equality test was not passed.

Administration of SIM (5 and 10 μM) alone (Figure 1A) or in combination with ALN (Figure 1B) contributed, in a dose dependent manner, to significantly reduced scratch closures. After 48 h the scratch exposed to SIM 5 μM alone had a closure of 73% ± 7% compared to the control and 75% ± 7% when exposed to SIM 5 μM combined with ALN (*p* = 0.001, respectively). SIM concentration of 10 μM resulted in even less closure, SIM 10 μM alone only closing 57% ± 16% of the initial scratch area after 48 h and 55% ± 9% when combined with ALN (*p* = 0.001, respectively compared to control). Administration of ALN alone induced no significant changes in the cell behavior, and the area of the scratch closely follows the same rate as the control. The same was observed with SIM 1 μM both alone and combined with ALN.

**Figure 2:**
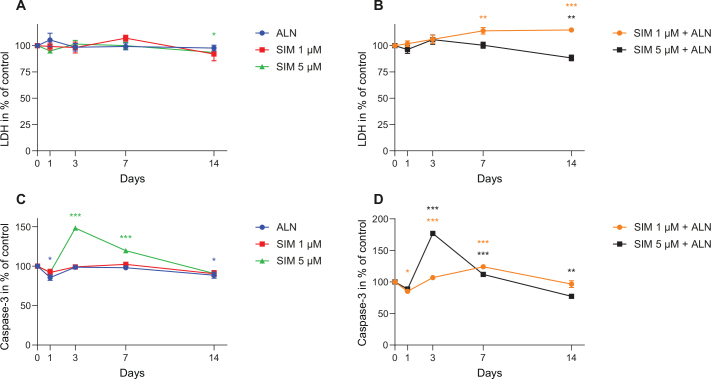
LDH activity measured in supernatants from fibroblast exposed to SIM (1 or 5 μM) individually (A) or in combination with ALN (B). Caspase-3 concentration secreted by fibroblasts exposed to SIM (1 or 5 μM) and ALN (5 μM) individually (C) or in combination (D) for 24 h prior to the harvest of medium. Significantly different results from control are indicated with ***p < 0.001, **p < 0.01 and *p < 0.05, analyzed by t-test or Mann–Whitney Rank Sum Test when normality and equality tests were not passed.

LDH activity measured in the media from the fibroblasts exposed to SIM 5 μM combined with ALN was slightly reduced at day 14 (Figure 2B). This contrasted with SIM 1 μM in combination with ALN, where LDH was slightly enhanced at day 14 (Figure 2B).

SIM 5 μM alone and in combination with ALN induced an increase in the Caspase-3 concentration in the cell culture media to 148% ± 1% and 177% ± 4% (*p* < 0.001, respectively) at day 3 (Figure 2C and D). SIM 1 μM combined with ALN induced an increase to 124% ± 3% (*p* < 0.001) at day 7 (Figure 2D), whereas SIM 1 μM and ALN alone had little or no effect on the secretion of Caspase-3 (Figure 2C).

### Secretion of factors affecting inflammation

**Figure 3:**
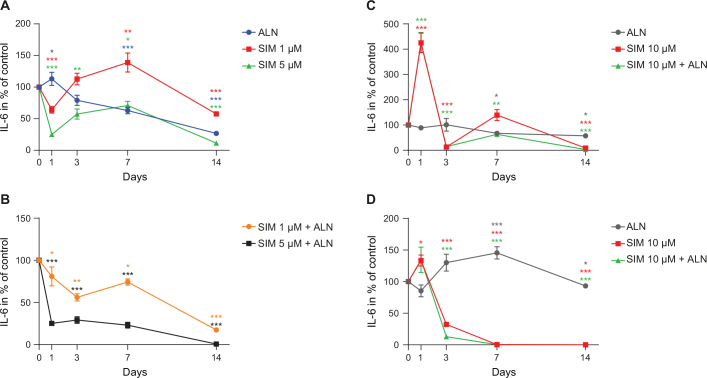
IL-6 concentration secreted by fibroblast exposed to SIM (1 or 5 μM) and ALN (5 μM) alone (A) or combined (B). IL-6 concentration from fibroblasts exposed to SIM 10 μM and ALN alone or combined (C). IL-6 concentration from osteoblasts exposed to SIM 10 μM alone or combined with ALN (D). Significantly different results from control are indicated with ***p < 0.001, **p < 0.01 and *p < 0.05, analyzed by t-test or Mann–Whitney Rank Sum Test when normality and equality tests were not passed.

There was a reduction in the amount of IL-6 secreted by fibroblasts when exposed to either ALN or SIM 5 μM alone or in combination (Figure 3A and B). When fibroblasts were incubated with SIM 10 μM, both alone and in combination with ALN, the concentration of IL-6 spiked at day 1 to 425% ± 7% and to 427% ± 10% (*p* < 0.001, respectively), prior to a drastic reduction to 13% ± 1% and 15% ± 1% of control (*p* < 0.001, respectively) at day 3 (Figure 3C).

An increase followed by a sharp decrease in IL-6 secretion was also observed for osteoblasts when incubated with SIM 10 μM alone or in combination with ALN (Figure 3D).

**Figure 4:**
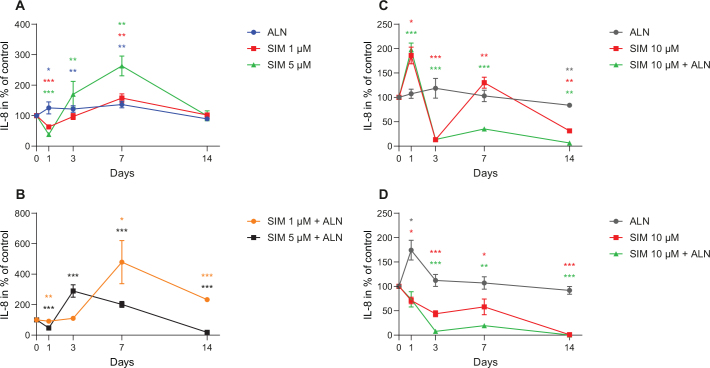
Measured IL-8 concentration secreted by fibroblast exposed to SIM (1 or 5 μM) alone (A) or in combination with ALN (5 μM) (B). IL-8 concentration secreted by fibroblasts exposed to SIM 10 μM, alone or combined with ALN (5 μM) (C). IL-8 concentration secreted by osteoblasts exposed to SIM 10 μM alone or combined with ALN (5 μM) (D). Significantly different results from control are indicated with ***p < 0.001, **p < 0.01 and *p < 0.05, analyzed by t-test or Mann–Whitney Rank Sum Test when normality and equality tests were not passed.

SIM (1–5 μM) alone or in combination with ALN caused an increase in IL-8 concentration secreted by fibroblasts (Figure 4A and B). SIM 5 μM alone and combined with ALN caused a reduction in concentration at day 1 before an increase was seen, whereas SIM 1 μM combined with ALN induced a concentration of 479% ± 239%. When fibroblasts were incubated with SIM 5 μM, the IL-8 concentration was the highest at day 7, however, when combined with ALN IL-8 peaked at day 3.

IL-8 concentration secreted from fibroblasts exposed to SIM 10 μM combined with ALN had an acute increase at day 1 before decreasing to 14% (19.2 pg/ml ± 0.2 pg/ml) of control (Figure 4C). When fibroblasts were exposed to SIM 10 μM the same was seen, but the IL-8 concentration rose to a level close to control at day 7 before sinking to 31.4% ± 1.0% of control (Figure 4C).

The osteoblast medium had a decrease in IL-8 concentration from day 1 when administrated with SIM 10 μM both alone and in combination with ALN, where the combination seemed to cause a more rapid decrease (Figure 4D).

**Figure 5:**
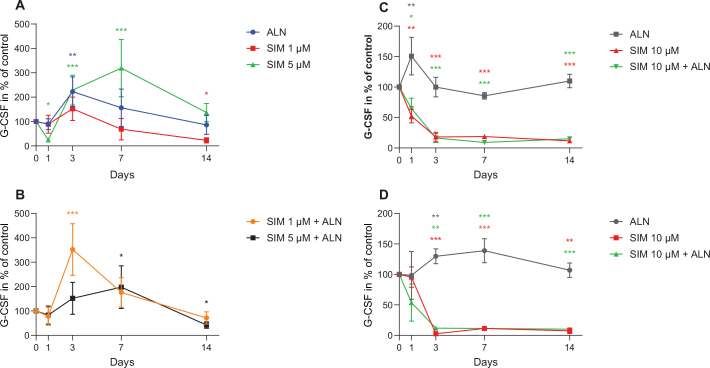
G-CSF concentration secreted by fibroblast after exposure to SIM (1 or 5 μM) alone (A) or in combination with ALN (5 μM) (B). G-CSF concentration secreted by fibroblast exposed to SIM 10 μM alone or combined with ALN (5 μM) (C). G-CSF concentration secreted by osteoblasts exposed to SIM 10 μM alone or combined with ALN (5 μM) (D). Significantly different results from control are indicated with ***p < 0.001, **p < 0.01 and *p < 0.05, analyzed by t-test or Mann–Whitney Rank Sum Test when normality and equality tests were not passed.

The amount of G-CSF secreted from fibroblasts after exposure to SIM 5 μM was reduced at day 1 followed by an increase to 319% ± 58.7% (*p* < 0.001) of control at day 7 (Figure 5A). An increase was also seen for SIM 1 μM combined with ALN at day 3 (Figure 5B). When fibroblasts were exposed to SIM 10 μM, alone and combined with ALN, a lasting reduction in G-CSF concentration was seen from day 1 (Figure 5C). Osteoblasts administered with SIM 10 μM alone and combined with ALN produced a similar reduction (Figure 5C and D).

**Figure 6:**
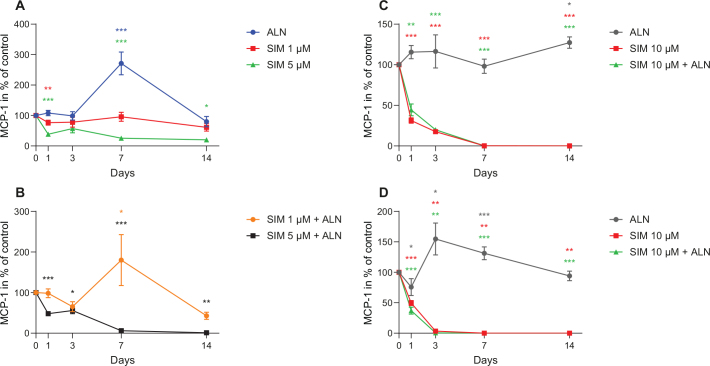
MCP-1 concentration in medium from fibroblasts after exposure to SIM (1 or 5 μM) alone (A) or in combination with ALN (5 μM) (B). MCP-1 concentration in medium from fibroblasts exposed to SIM 10 μM alone or combined with ALN (5 μM) (C). MCP-1 concentration in medium from osteoblast exposed to SIM 10 μM alone or combined with ALN (5 μM) (D). Significantly different results from control are indicated with ***p < 0.001, **p < 0.01 and *p < 0.05, analyzed by t-test or Mann–Whitney Rank Sum Test when normality and equality tests were not passed.

The MCP-1 levels secreted by fibroblasts exposed to SIM (5–10 μM), individually or combined with ALN, were significantly reduced compared to control at most time points evaluated (Figure 6A–C). SIM 5 μM combined with ALN induced the lowest concentration (1.1% of control) at day 14 (*p* = 0.008) (Figure 6B). Both ALN and SIM 1 μM combined with ALN caused a significantly increased concentration at day 7, which was not seen for any of the other exposures (Figure 6A and B). Administration of SIM 10 μM, alone or combined with ALN, abolished the MCP-1 secretion from fibroblasts compared to control, with the lowest concentration being < 1% of control (0.7 pg/ml ± 0.4 pg/ml for SIM 10 μM alone, and 1.5 pg/ml ± 1.5 pg/ml for SIM 10 μM combined with ALN) at day 14 (*p* < 0.001, respectively) (Figure 6C). The secretion of MCP-1 from osteoblasts showed a similar pattern, with both SIM 10 μM alone and combined with ALN being at the lowest level at day 7 (Figure 6D).

**Figure 7:**
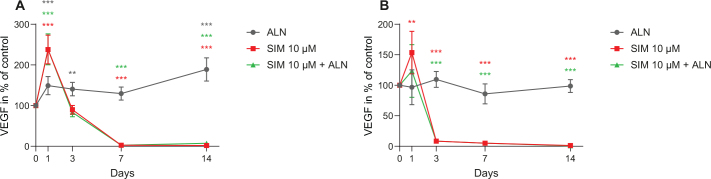
VEGF concentration secreted by fibroblasts exposed to SIM (10 μM) alone or combined with ALN (5 μM) (A). VEGF concentration secreted by osteoblasts exposed to SIM (10 μM) alone or combined with ALN (5 μM) (B). Results are presented as % of control and significantly different results from control are indicated with ***p < 0.001, **p < 0.01 and *p < 0.05, analyzed by t-test or Mann–Whitney Rank Sum Test when normality and equality tests were not passed.

When fibroblasts were exposed to SIM 10 μM, alone and combined with ALN, the VEGF concentrations in the cell culture media were increased to 238% ± 4% and 238% ± 25% of control at day 1 (*p* < 0.001, respectively) (Figure 7A). However, at day 7 the levels were both reduced to 3% ± < 1% (3.0 pg/ml ± 0.3 pg/ml, respectively) (*p* < 0.001, respectively). The VEGF levels secreted by osteoblasts after administration of SIM 10 μM were also increased at day 1, to 154% ± 0.0% (*p* = 0.005) (Figure 7B). At day 7, osteoblasts incubated with SIM 10 μM, alone and combined with ALN, resulted in a lasting decrease in VEGF concentration staying at 5% ± 0%.

In the cellular experiments with SIM concentrations (1–5 μM) the levels of VEGF in cell culture media were below detection limit of the assay for all samples tested.

## Discussion

This study demonstrated that high concentrations of simvastatin (5–10 μM) caused a reduction in migration and the secretion of cytokine/chemokines (IL-6, IL-8, G-CSF, MCP-1 and VEGF) from confluent fibroblasts. The observed effects were almost identical between fibroblasts exposed to the highest concentrations of SIM alone and those exposed to SIM in combination with ALN. Administration of SIM and ALN to osteoblasts induced the same pattern, indicating a SIM-concentration dependent reduction in the secretion of cytokines after 3–14 days incubation, as this pattern was not seen for SIM 1 μM, either alone or combined with ALN.

Both SIM and ALN have been reported to have positive effects on bone remodeling and growth [8, 10, 14], whereas there are few reports on the combined effects of statins and bisphosphonates [15, 16].

Several articles point to SIMs positive effect on inflammation and bone health, by stimulating wound healing and the formation of bone tissue [7, 11, 12]. The observed reduction in the secretion of IL-6 and IL-8 from both fibroblasts and osteoblasts may support the observed positive effects on inflammation and stimulation of repair processes [17–19]. Although both IL-6 and IL-8 are recognized to promote pro-inflammatory activity, which has been established in various models, they have also been found to stimulate differentiation and growth processes [20, 21].

The secretion of VEGF, an essential growth factor in angiogenesis [22], was almost abolished by administration of SIM alone and in combination with ALN, indicating that both fibroblasts and osteoblasts reduce angiogenic signals upon exposure to SIM at concentrations of 5 μM or higher. IL-6 [23], IL-8 [24], and MCP-1 [25] are also found to induce angiogenesis. The observed reduction of all these factors, related to processes that are characteristic of angiogenesis, may lead to reduced remodeling, repair, and growth as angiogenesis is a key component in bone repair [26, 27] and periodontal remodeling [28].

In combination with angiogenesis, migration of cells is needed to heal a wound. Earlier studies have shown that there was a reduction in fibroblast migration when 10 μM of SIM was administered [13, 29]. SIM disrupts the cell signaling network that regulates the actin cytoskeleton dynamic, which again might affect the response of gingival mesenchymal cells during wound healing [13]. The observed dose dependent reduction in scratch closure might be explained by this mechanism. A high concentration of SIM may therefore cause reduced wound healing both by reducing factors connected to angiogenesis, as well as reducing fibroblast migration.

The effects observed in this *in vitro* study might not reflect the *in vivo* situations. Cells grown in 2D cell cultures adhere to the plastic surface of the plate, receiving maximum exposure to drugs with optimal diffusion of nutrients and waste products [30, 31]. *In vivo*, cells are exposed to gradients of nutrients, drugs, and more in a 3D microenvironment with other cells [32, 33]. Positive effects on bone, like preventing osteoporosis and aiding in fracture and bone defect healing, caused by SIM are observed at high dosages (20 mg/kg/day) [9]. As statins have limited bone affinity, systematic administration likely requires a much higher dosage than local administration to cause a similar effect [8].

It has been speculated that high doses of SIM could cause hazardous adverse effects [8]. Two reports have presented cases where long term use of high dose (40 mg daily) SIM seem to be the sole cause of ONJ [34, 35]. ONJ is typically presented as a nonhealing extraction site, surrounded by enflamed soft tissue [36]. A reduction in factors linked to angiogenesis, as well as reduced wound closure as seen in the wound scratch assay, caused by SIM could have a negative impact on healing. As ALN has ONJ as an established side effect, though with a very low chance of it developing [37, 38], combination of long-term high dose SIM with ALN could possibly increase the chances of such unwanted side effects.

The concentrations used in these *in vitro* experiments are based upon concentrations used in earlier *in vitro* studies [7, 13].

It cannot be ruled out that some of the observed effects may be due to cytotoxicity or cell death, although the LDH levels after exposure to high levels of ALN [4] or SIM were not much different from those secreted by the unexposed control cells. There was not always agreement between the measured LDH activity and the caspase-3 concentrations in the cell culture media at the various time points tested. Increased LDH activity in the medium indicates cell death by apoptosis or necrosis, while increased caspase-3 concentrations is a hallmark of apoptosis [39]. Although some report a relationship between these two markers *in vivo* [40], a reduction in LDH activity has been shown to induce apoptosis in other cell types *in vitro* [41].

## Conclusion

In contrast to higher concentrations, lower concentrations of SIM alone and combined with ALN do not appear to have a negative impact on cellular responses in fibroblast. Higher concentrations of SIM alone or in combination with ALN, on the other hand, affected the secretion of factors involved in angiogenesis in both fibroblasts and osteoblasts, which in turn may alter the microenvironment in a tissue, and thus influence wound healing. This might contribute to adverse local side effects, such as ONJ. More research is needed to fully understand the combined effect of simvastatin and alendronate.
